# A context-independent *N*-glycan signal targets the misfolded extracellular domain of *Arabidopsis* STRUBBELIG to endoplasmic-reticulum-associated degradation

**DOI:** 10.1042/BJ20141057

**Published:** 2014-12-05

**Authors:** Silvia Hüttner, Christiane Veit, Ulrike Vavra, Jennifer Schoberer, Martina Dicker, Daniel Maresch, Friedrich Altmann, Richard Strasser

**Affiliations:** *Department of Applied Genetics and Cell Biology, University of Natural Resources and Life Sciences, Muthgasse 18, A-1190 Vienna, Austria; †Department of Chemistry, University of Natural Resources and Life Sciences, Muthgasse 18, A-1190 Vienna, Austria

**Keywords:** cell biology, endoplasmic reticulum, endoplasmic-reticulum-associated degradation (ERAD), glycobiology, glycoprotein, glycosylation, protein degradation, protein misfolding, BRI1, brassinosteroid insensitive 1, CNX/CRT, calnexin/calreticulin, CPY*, mutant variant of yeast carboxypeptidase Y, Endo H, endoglycosidase H, ER, endoplasmic reticulum, ERAD, ER-associated degradation, ERQC, ER quality control, mRFP, monomeric RFP, MRH, mannose 6-phosphate receptor homology, MS, Murashige and Skoog, PDI, protein disulfide isomerase, PGC, porous graphitic carbon, PNGase, peptide-*N*-glycosidase, RIPA, radio immunoprecipitation assay, SUB, STRUBBELIG, SUBEX, STRUBBELIG extracellular domain, ΔXTFT, *Nicotiana benthamiana* glycosylation mutant deficient in β1,2-xylosyltransferase and core α1,3-fucosyltransferase

## Abstract

*N*-glycosylation of proteins plays an important role in the determination of the fate of newly synthesized glycoproteins in the endoplasmic reticulum (ER). Specific oligosaccharide structures recruit molecular chaperones that promote folding or mannose-binding lectins that assist in the clearance of improperly-folded glycoproteins by delivery to ER-associated degradation (ERAD). In plants, the mechanisms and factors that recognize non-native proteins and sort them to ERAD are poorly understood. In the present study, we provide evidence that a misfolded variant of the STRUBBELIG (SUB) extracellular domain (SUBEX-C57Y) is degraded in a glycan-dependent manner in plants. SUBEX-C57Y is an ER-retained glycoprotein with three *N*-glycans that is stabilized in the presence of kifunensine, a potent inhibitor of α-mannosidases. Stable expression in *Arabidopsis thaliana* knockout mutants revealed that SUBEX-C57Y degradation is dependent on the ER lectin OS9 and its associated ERAD factor SEL1L. SUBEX-C57Y was also stabilized in plants lacking the α-mannosidases MNS4 and MNS5 that generate a terminal α1,6-linked mannose on the C-branch of *N*-glycans. Notably, the glycan signal for degradation is not constrained to a specific position within SUBEX-C57Y. Structural analysis revealed that SUBEX-C57Y harbours considerable amounts of Glc_1_Man_7_GlcNAc_2_ *N*-glycans suggesting that the ER-quality control processes involving calnexin/calreticulin (CNX/CRT) and ERAD are tightly interconnected to promote protein folding or disposal by termination of futile folding attempts.

## Abstract 2

The misfolded extracellular domain of a specific leucine-rich repeat receptor-like kinase from *Arabidopsis thaliana* serves as a novel plant ERAD substrate that is degraded in a glycan-dependent way by the OS9–SEL1L–HRD1 complex.

## INTRODUCTION

The endoplasmic reticulum (ER) is the major site for protein folding and maturation in the secretory pathway of eukaryotic cells. Membrane-anchored and luminal proteins are folded and assembled before they are secreted or directed to their final destination within the cells. The ER of all eukaryotes has an elaborate quality control [ER quality control (ERQC)] system that promotes proper folding and assembly of proteins. Failure to acquire a native conformation results in the absence of the functional protein from its designated cellular compartment, unwanted protein aggregation or secretion of defective proteins that might endanger the cellular protein homoeostasis and ultimately the biological function of the cells and the organisms. Consequently, these aberrant proteins are cleared from the ER by a process known as ER-associated degradation (ERAD) [1–3].

ERAD can be divided into distinct steps, including recognition of the misfolded protein, transport and translocation of the ERAD substrate into the cytosol and finally degradation by the proteasome. Since the fidelity of secretory proteins is crucial for the survival of eukaryotic cells, most ERQC and ERAD components are highly conserved between species. Most of our knowledge about ERAD and the involved protein machinery come from studies in *Saccharomyces cerevisiae* and mammalian cells. In recent years, however, the importance of ERQC and ERAD in different cellular processes, including salt stress tolerance and immune response during pathogen defence, has been recognized for plants [4–6], and proteins with similarity to different yeast and mammalian ERAD components have been characterized [7–11].

Many proteins synthesized in the ER are *N*-glycosylated and the oligosaccharides that are co-translationally attached have important functions during protein maturation [12]. *N*-glycans not only influence the folding process, for example by steric hindrance or through subjecting polypeptides to the calnexin/calreticulin (CNX/CRT) cycle, but also function as signals that direct misfolded glycoproteins towards degradation [13]. An exposed terminal α1,6-linked mannose generated by class I α-mannosidases in the ER serves as a degradation signal during ERAD of defective glycoproteins in *S. cerevisiae* [14]. This specific oligosaccharide structure recruits the mannose binding lectin YOS9 and associated factors like HRD3 (yeast orthologue of human SEL1L) that direct ERAD substrates to the site of dislocation. Mannose removal plays a similar role in disposal of glycoproteins in mammalian cells and distinct ERAD branches have been defined that employ overlapping sets of components to account for structurally diverse sets of substrates [15].

In plants, comparatively little is known about the different requirements for ERAD of various substrate classes including glycosylated and non-glycosylated proteins, as well as pathways for luminal and membrane-bound proteins. The luminal catalytic A chain of ricin, for example, is glycosylated and degraded by ERAD, but its disposal occurs by an alternative *N*-glycan-independent mechanism [16,17]. Studies on *N*-glycan-dependent ERAD pathways in plants have been focused mainly on mutant variants of *Arabidopsis thaliana* brassinosteroid insensitive 1 (BRI1) [18,19] which is a heavily glycosylated transmembrane receptor involved in brassinosteroid signalling [20]. Despite some recent progress in structural characterization of the *N*-glycans from the ERAD substrate BRI1–5, the presence of 14 potential *N*-glycans make a systematic analysis of their role in glycan-dependent ERAD very challenging [11]. Consequently, further progress in the understanding of the glycan-dependent processes during ERAD in plants is hampered by the absence of suitable glycoprotein substrates.

In the present study, we examined the requirements for degradation of a topologically different glycoprotein substrate with a reduced number of *N*-glycans. The *A. thaliana* STRUBBELIG (SUB) protein is a cell surface leucine-rich repeat receptor-like kinase that plays a role in tissue morphogenesis of different plant organs [21]. In a previous study, a number of sub-mutants were identified and their effect on the SUB protein structure and function was characterized [22]. A GFP-tagged mutant variant, which has a cysteine to tyrosine mutation at position 57 (SUB-C57Y) in the extracellular domain, appears to be degraded in a glycan-dependent way because the fluorescence signal in roots was increased upon treatment with the specific class I α-mannosidase inhibitor kifunensine [22]. However, this finding was not confirmed at the protein level and the role of *N*-glycosylation in SUB-C57Y degradation, as well as the putative mechanism and the nature of involved ERAD components, were not further investigated. In the present study, we expressed the glycosylated extracellular domain of SUB-C57Y (called SUBEX-C57Y) in *Nicotiana benthamiana* and *A. thaliana* plants and found that this misfolded protein is subjected to ERAD in a glycan-dependent manner. In addition, we performed site-directed mutagenesis to generate hypoglycosylated SUBEX-C57Y and determined the contribution of individual *N*-glycans to degradation. SUBEX-C57Y is a novel glycoprotein ERAD substrate that allows dissecting of the glycan-dependent steps for selective degradation of misfolded proteins in plants.

## EXPERIMENTAL

### Plant materials

*A. thaliana* plants (mutants and the Col-0 ecotype which was used as a wild-type control) were grown under long-day conditions at 22°C as described previously [23]. T-DNA insertion lines *os9-1* (SALK_029413), *sel1l* (SALK_109430), *mns4-1* (SALK_119093), *mns5-1* (GT5_84786) and the double mutant *mns4-1 mns5-1* were obtained from the European *Arabidopsis* Stock Centre or by crossing, respectively, and were described in more detail recently [10,11]. For treatment with kifunensine and cycloheximide, *A. thaliana* seedlings grown on solid 0.5× Murashige and Skoog (MS) medium containing 1% sucrose were harvested and incubated in liquid 0.5× MS medium supplemented with 1% sucrose and 50 μM kifunensine (Sigma–Aldrich) and/or 100 μg/ml cycloheximide (Sigma–Aldrich). *N. benthamiana* plants were grown on soil under long-day conditions (8 h light/16 h dark) at 24°C.

### Plasmid construction and generation of transgenic plants

To create vectors expressing SUBEX and SUBEX-C57Y, the N-terminal extracellular domain (amino acids 1–341) of SUB (*A. thaliana* gene locus: At1g11130) was PCR amplified from Col-0 cDNA with the primers SUB-1F (TTCTAGAATGAGCTTTACAAGATGGGAAGTG)/-2R (TGGATCCTCTTTGAGTGGACCAGAATTTTCC) and subcloned into pCR4 Blunt TOPO vector (Life Technologies). The C57Y mutation for SUBEX-C57Y was introduced into TOPO-SUBEX by using the QuikChange site-directed mutagenesis kit (Agilent Technologies) with the primers SUB-4F, TTTGGAGGAGACCCTTATGGAGAAAAGTGGCAA and SUB-4R, TTGCCACTTTTCTCCATAAGGGTCTCCTCCAAA. SUBEX and SUBEX-C57Y, respectively, were then ligated into the XbaI/BamHI digested plasmids p20F [24] and p47 (like p20F but with a UBQ10 promoter instead of a CaMV35S promoter and a hygromycin resistance gene for selection of transgenic plants) resulting in the plant expression vectors p20-SUBEX (CaMV35S:SUBEX-GFP), p20-SUBEX-C57Y (CaMV35S:SUBEX-C57Y-GFP), p47-SUBEX (UBQ10:SUBEX-GFP) and p47-SUBEX-C57Y (UBQ10:SUBEX-C57Y-GFP). The SUBEX-C57Y mutants missing one (‘NQ1’, ‘NQ2’, ‘NQ3’), two (‘NQ12’, ‘NQ13’, ‘NQ23’) or all three (‘NQ123’) *N*-glycosylation sites were constructed by performing site-directed mutagenesis using p20-SUBEX-C57Y as template. The respective single *N*-glycosylation mutants were generated using the following primers: N70Q (NQ1), SUBNQ-1F, GTGGTGTGTGACTCCTCACAGATCACAGAAATAAGGATAC and SUBNQ-1R, GTATCCTTATTTCTGTGATCTGTGAGGAGTCACACACCAC; N119Q (NQ2), SUBNQ-2F, CTTCTTCCATCCGACAGCTATCTCTCTCAAGCAATCGC and SUBNQ-2R, GCGATTGCTTGAGAGAGATAGCTGTCGGATGGAAGAAG; and N243Q (NQ3), SUBNQ-3F, GGAACTCCGTTCCAGACATCGATTATAACACCACC and SUBNQ-3R, GGTGGTGTTATAATCGATGTCTGGAACGGAGTTCC. The construction of the plasmids p31-AtOS9 [CaMV35S:OS9-mRFP (monomeric RFP)], p31-AtOS9R201A (CaMV35S:OS9R201A-mRFP) and p60-SEL1L (CaMV35S:SEL1L-HA) used in co-immunoprecipitation experiments, were described previously [10]. For stable expression, *A. thaliana* mutant and wild-type plants were floral dipped with *Agrobacterium tumefaciens* strain UIA143 containing p20-/p47-SUBEX-C57Y or p47-SUBEX-C57Y NQ123 and transgenic plants were later selected on MS plates containing 50 μg/ml kanamycin or hygromycin respectively.

### Subcellular localization of SUBEX/SUBEX-C57Y

For subcellular localization studies, agrobacterium strain UIA143 carrying p20-SUBEX or p20-SUBEX-C57Y was diluted to a *D*_600_ of 0.1 and expressed in leaf epidermal cells of 5-week-old *N. benthamiana* plants following agrobacterial leaf infiltration [24]. Sections of infiltrated leaves were analysed 24 h after infiltration on a Leica TCS SP2 confocal microscope, as described previously [24]. For co-localization experiments p20-SUBEX and p20-SUBEX-C57Y were infiltrated together with the ER-marker OST4A-mRFP (*D*_600_ of 0.05), which contains the OST4 subunit of the *A. thaliana* oligosaccharyltransferase complex [25] or the Golgi marker MNS1-mRFP (*D*_600_ of 0.05), which harbours *A. thaliana* Golgi-α-mannosidase I [26].

### Co-purification of transiently-expressed ERAD components

Leaves of 5-week-old *N. benthamiana* plants were co-infiltrated (*D*_600_ of 0.3) with agrobacteria carrying p20-SUBEX, p20-SUBEX-C57Y and either p31-AtOS9, p31-AtOS9R201A, p60-SEL1L, with or without 20 μM kifunensine. 1 g of leaf material was harvested 24 h after infiltration and proteins were extracted with 2 μl/mg radio immunoprecipitation assay (RIPA) buffer [50 mM Tris/HCl (pH 7.5), 150 mM NaCl, 0.1% (w/v) SDS, 0.5% (w/v) sodium deoxycholate, 1% (v/v) Triton X-100, 1 mM PMSF] supplemented with 1% (v/v) protease inhibitor cocktail (Sigma-Aldrich). The extract was centrifuged twice (10000 ***g*** for 10 min at 4°C; 13000 ***g*** for 30 min at 4°C), the cleared supernatant diluted with 1 volume of dilution buffer [10 mM Tris/HCl (pH 7.5), 150 mM NaCl, 0.5 mM EDTA, 1 mM PMSF] and incubated with 25 μl of GFP trap beads (Chromotek) for 1.5 h at 4°C on a rocking shaker. After an extensive washing step with the dilution buffer, the protein fractions were eluted with SDS/PAGE loading buffer by incubation for 5 min at 95°C. Subsequently, the samples were separated by SDS/PAGE (7%) followed by immunoblot analysis using anti-GFP-HRP (Miltenyi Biotec), anti-mRFP (Chromotek) and anti-HA (Roche) antibodies.

### Enzymatic deglycosylation

Proteins were extracted from infiltrated *N. benthamiana* leaves 24–36 h after infiltration with 1 μl/mg RIPA buffer. After two centrifugation steps (10000 ***g*** for 10 min at 4°C; 13000 ***g*** for 30 min at 4°C) 3× SDS/PAGE loading buffer was added to the cleared supernatant and the proteins were incubated with or without endoglycosidase H (Endo H) and peptide-*N*-glycosidase F (PNGase F) (both from New England Biolabs) according to the manufacturer's manual.

### Incubation with mannosidase and proteasome inhibitors

Small leaf segments of *N. benthamiana* or *A. thaliana* seedlings expressing SUBEX-GFP or SUBEX-C57Y-GFP were incubated in liquid 0.5× MS medium supplemented with 1% sucrose and with or without 50 μM kifunensine, 100 μM clasto-lactacystin β-lactone (Sigma-Aldrich) or 50 μM MG132 (Sigma-Aldrich) for 5 h in the dark. Subsequently, proteins were extracted with 1× PBS containing 1% Triton X-100 and 1% protease inhibitor cocktail and subjected to SDS/PAGE and immunoblotting with anti-GFP-HRP, anti-ubiquitin (P4D1, Santa Cruz Biotechnology) and anti-protein disulfide isomerase (PDI) [27] antibodies. Detection was done with a ChemiDoc imager (Bio-Rad Laboratories) and quantification with Quantity One software (Bio-Rad Laboratories).

### Glycan analysis

For LC-ESI-MS analysis of glycopeptides, SUBEX-GFP and SUBEX-C57Y-GFP were transiently expressed in *N. benthamiana* leaf epidermal cells and purified using GFP trap beads as described above. The purified proteins were separated by SDS/PAGE (10%) and stained with Coomassie Brilliant Blue. The bands corresponding to SUBEX-GFP and SUBEX-C57Y-GFP were excised from the gel, destained, carbamidomethylated, in-gel trypsin digested and analysed by LC-ESI-MS as described in detail previously [28]. For isomer analysis, glycans were removed from SUBEX or SUBEX-C57Y by PNGase A digestion and subsequently analysed by porous graphitic carbon LC-ESI (PGC)-LC-ESI-MS as described in detail previously [29].

## RESULTS

### SUBEX-C57Y is retained in the ER

**Figure 1:**
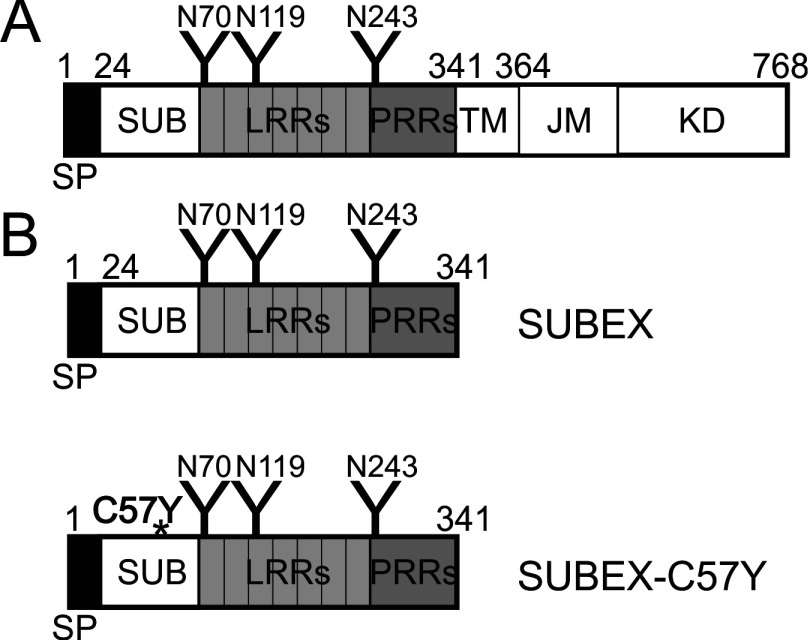
Domain structure of SUB and SUBEX variants (**A**) Schematic representation of the full-length SUB protein. Different protein domains are indicated [22]. (**B**) Schematic representation of the truncated proteins SUBEX and SUBEX-C57Y, comprising only the extracellular domain of SUB. The C57Y mutation is indicated by an asterisk. *N*-glycosylation sites are represented by ‘Y’ and their amino acid positions are shown. JM, juxtamembrane domain; KD, kinase domain; LRR, leucine-rich repeat; PRR, proline-rich repeat; SP, signal peptide; SUB, SUB-domain; TM, transmembrane domain.

To generate a soluble glycoprotein substrate for the study of the glycan-dependent ERAD pathway in plants, we expressed a variant (called SUBEX) of SUB lacking its transmembrane and intracellular kinase domain in plants (Figure 1A). To this end, we amplified the coding region for the N-terminal 341 amino acids from cDNA of *A. thaliana* wild-type and the C57Y mutant (SUBEX-C57Y) variant was obtained by site-directed mutagenesis. SUBEX-C57Y harbours a signal peptide, the leucine- and proline-rich repeat domains and all three predicted *N*-glycosylation sites from SUB (Figure 1B) [22].

**Figure 2:**
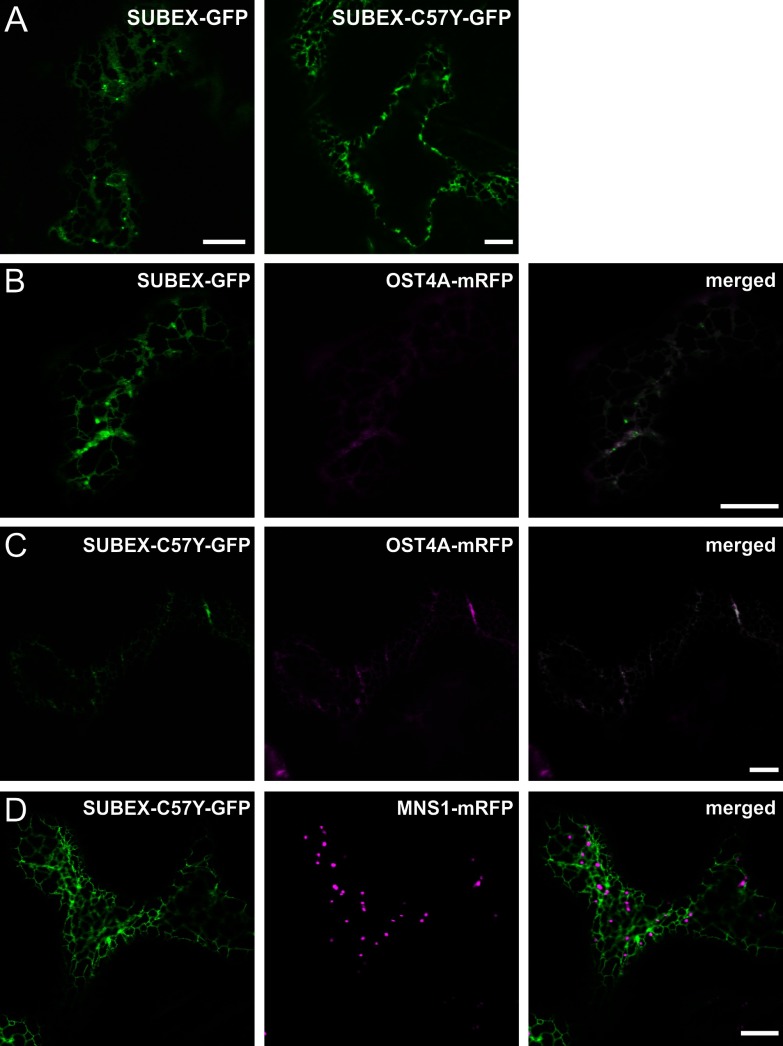
SUBEX and SUBEX-C57Y display ER-localization (**A**) SUBEX-GFP and SUBEX-C57Y-GFP respectively were transiently expressed in *N. benthamiana* leaf epidermal cells and analysed by confocal microscopy. (**B**) Expression of SUBEX-GFP (green) together with the ER-marker OST4A-mRFP (magenta) showing co-location (merged image). (**C**) Expression of SUBEX-C57Y-GFP (green) together with the ER-marker OST4A-mRFP (magenta) showing co-location (merged image). (**D**) Co-expression of SUBEX-C57Y-GFP (green) and the Golgi-marker MNS1-mRFP (magenta) indicates specific ER-localization of SUBEX-C57Y-GFP. Scale bars=10 μm.

To monitor their expression and subcellular localization, we generated plant expression constructs, where GFP was fused to the C-terminal end of SUBEX and SUBEX-C57Y and the resulting SUBEX-GFP and SUBEX-C57Y-GFP proteins were transiently expressed in *N. benthamiana* leaf epidermal cells by agrobacterial infiltration. Analysis of their subcellular location by confocal laser-scanning microscopy revealed that both variants show a reticulate fluorescence signal, indicating SUBEX-GFP and SUBEX-C57Y-GFP accumulation in the ER (Figure 2A). This finding was confirmed by co-expression of the ER-marker OST4A-mRFP [25] (Figures 2B and 2C). By contrast, no co-localization was observed for SUBEX-C57Y-GFP with the Golgi-marker MNS1-mRFP [23], indicating that SUBEX-C57Y is exclusively present in the ER (Figure 2D).

### SUBEX-C57Y is glycosylated with oligomannosidic *N*-glycans

**Figure 3:**
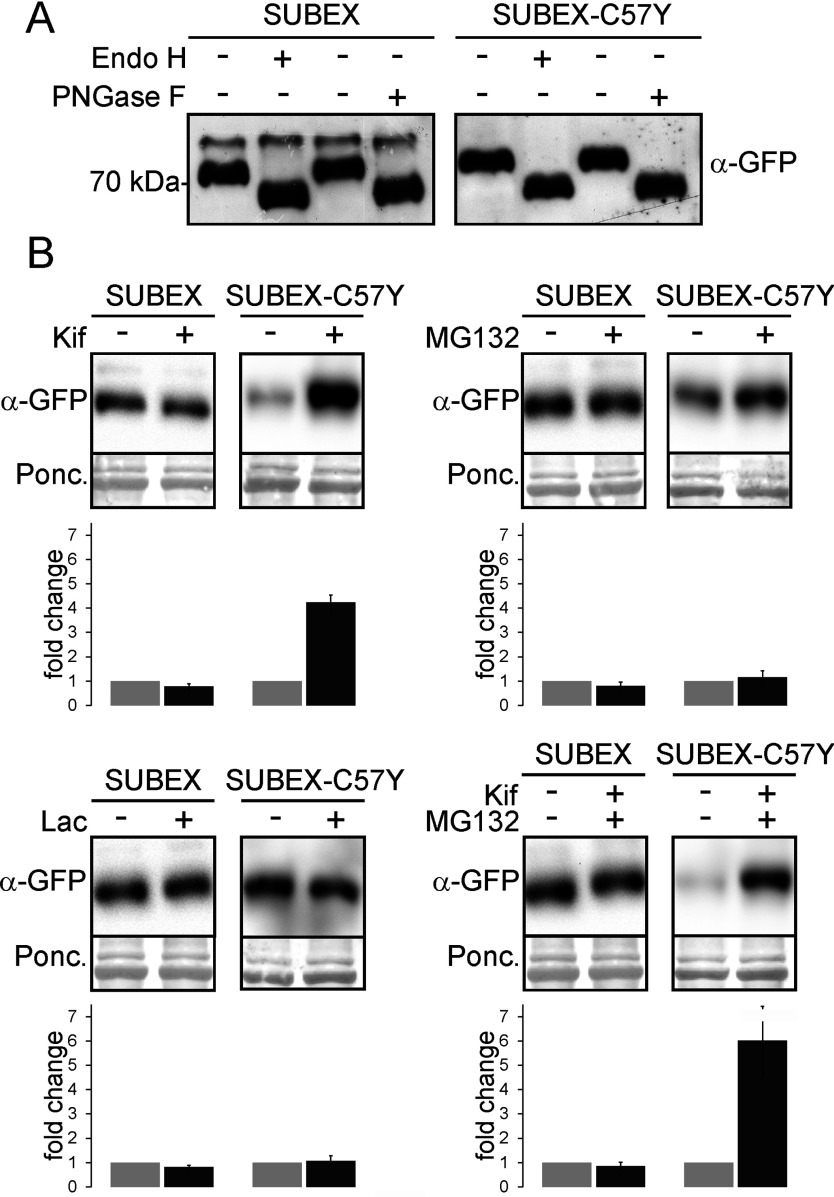
Stabilization of SUBEX-C57Y is glycan-dependent but proteasome-independent (**A**) SUBEX-GFP and SUBEX-C57Y-GFP were transiently expressed in *N. benthamiana* plants. Protein extracts from leaves were digested with Endo H and PNGase F and subjected to SDS/PAGE followed by immunoblotting. The shift upon endoglycosidase digestion shows the presence of oligomannosidic *N*-glycans. (**B**) Leaves expressing SUBEX-GFP or SUBEX-C57Y-GFP were incubated for 5 h with the α-mannosidase inhibitor kifunensine (Kif), the proteasome inhibitors MG132 or lactacystin (Lac) or a combination thereof. Proteins were extracted and analysed by SDS/PAGE followed by immunoblotting. Ponceau S (Ponc.) staining served as a loading control. At the bottom of each blot, the quantification of the SUBEX and SUBEX-C57Y signals are shown (mean of three independent experiments; error bars=S.D.). The amount of SUBEX or SUBEX-C57Y without inhibitor was set to 1 (light grey bars) and the relative stabilization of inhibitor-treated samples is shown (dark grey bars).

Three predicted *N*-glycosylation sites (N70, N119, N243) are present in the SUBEX amino acid sequence (Figure 1B). To investigate the presence and nature of the *N*-glycans, SUBEX-GFP and SUBEX-C57Y-GFP were transiently expressed in *N. benthamiana* leaves and total protein extracts subjected to digestion by Endo H and PNGase F. Since core α1,3-fucose on complex plant *N*-glycans interferes with PNGase F activity [30], *N. benthamiana* ΔXTFT (*N. benthamiana* glycosylation mutant deficient in β1,2-xylosyltransferase and core α1,3-fucosyltransferase) plants were used, which are almost completely devoid of core α1,3-fucose [31]. Both SUBEX and SUBEX-C57Y were sensitive towards Endo H and PNGase F digestions and no difference in mobility between samples treated with either of the two endoglycosidases could be detected by immunoblots (Figure 3A) indicating that SUBEX and SUBEX-C57Y are glycosylated mainly with oligomannosidic *N*-glycans as typical for ER-resident glycoproteins.

### SUBEX-C57Y is degraded in a glycan-dependent, but proteasome-independent, manner

To assess the stability of transiently expressed SUBEX and SUBEX-C57Y, we measured their accumulation upon treatment with kifunensine that blocks α-mannosidase activity and subsequently ERAD of glycoproteins in plants [11,18,23]. Incubation with kifunensine resulted in considerably increased SUBEX-C57Y protein levels, whereas levels of SUBEX were similar in the treated and untreated samples (Figure 3B). By contrast, treatment with the proteasome inhibitors MG132 or lactacystin had no effect on either SUBEX or SUBEX-C57Y accumulation. Taken together, these data indicate that SUBEX-C57Y is degraded in a α-mannosidase-dependent manner.

### Stabilization of SUBEX-C57Y by kifunensine is dependent on the presence of its *N*-glycans

**Figure 4:**
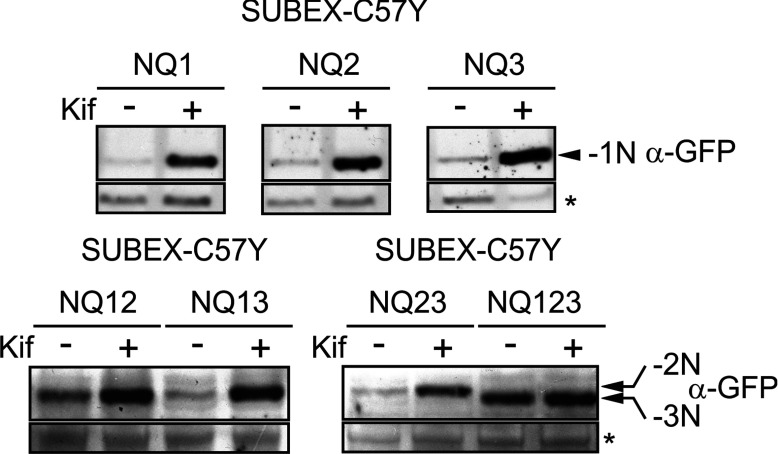
Stabilizing effect of kifunensine on SUBEX-C57Y is dependent on the presence of *N*-glycans Protein extracts from plants transiently expressing SUBEX-C57Y-GFP lacking one (NQ1, NQ2, NQ3), two (NQ12, NQ13, NQ23) or all three (NQ123) of its *N*-glycans were incubated with/without kifunensine (Kif) and subjected to SDS/PAGE followed by immunoblotting. The asterisk indicates an unspecific band used as loading control.

For some glycosylated ERAD substrates, like a mutant variant of yeast carboxypeptidase Y (CPY*), it has been shown that only the most C-terminal of the four *N*-glycans is required for ERAD [32,33]. To examine the contribution of individual *N*-glycans to SUBEX-C57Y stability, we eliminated *N*-glycosylation sites by changing asparagine to glutamine via site-directed mutagenesis. In this way, single mutants missing the first (N70, ‘NQ1’), second (N119, ‘NQ2’), third (N243, ‘NQ3’) *N*-glycosylation site or double and triple mutants missing two (‘NQ12’, ‘NQ13’, ‘NQ23’) or all three (‘NQ123’) sites were generated. The mutant variants were transiently expressed in *N. benthamiana* leaves and their accumulation in the presence of kifunensine was analysed. The single glycosylation-site mutants SUBEX-C57Y NQ1, NQ2 and NQ3 were considerably stabilized by kifunensine (Figure 4). Although similar results were obtained for the SUBEX-C57Y double glycosylation site mutants NQ12, NQ13 and NQ23, the fully unglycosylated SUBEX-C57Y variant NQ123 was stabilized in the absence of the α-mannosidase inhibitor and no further accumulation was observed in the presence of kifunensine. These data strongly suggest that the presence of a single *N*-glycan is necessary and sufficient for the glycan-dependent degradation of SUBEX-C57Y.

### Degradation of SUBEX-C57Y requires OS9, SEL1L and MNS4/MNS5

**Figure 5:**
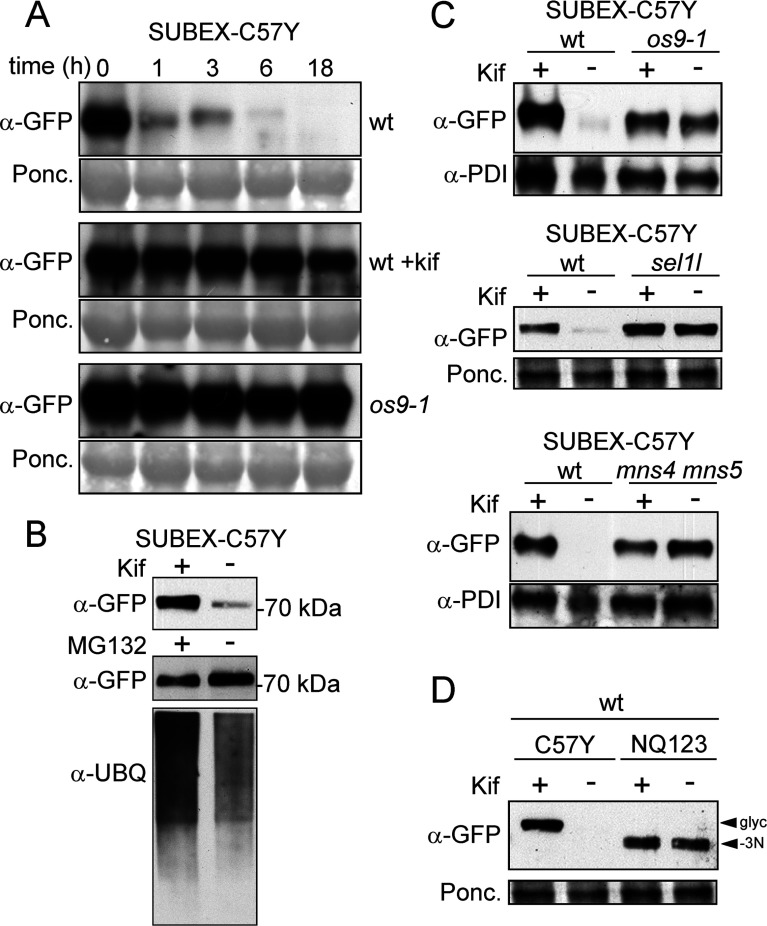
SUBEX-C57Y is stabilized in *A. thaliana os9-1*, *sel1l* and *mns4 mns5* mutants (**A**) Leaves from wild-type (wt) and *os9-1* plants stably expressing SUBEX-C57Y-GFP were incubated with/without kifunensine (Kif). At the indicated time-points, proteins were extracted and analysed by immunoblotting. (**B**) Seedlings from wt plants stably expressing SUBEX-C57Y-GFP were incubated with/without Kif or MG132. Protein extracts were subjected to SDS/PAGE and immunoblotting. Detection of ubiquitin serves as a control for MG132 treatment. (**C**) Leaves from *os9-1*, *sel1l* and *mns4 mns5* mutants stably expressing SUBEX-C57Y-GFP were incubated with or without Kif. (**D**) Leaves from wt plants stably expressing SUBEX-C57Y-GFP or the non-glycosylated SUBEX-C57Y-GFP variant NQ123 were incubated with/without Kif. Ponceau S (Ponc.) staining or anti-PDI detection served as loading controls.

*A. thaliana* OS9, an orthologue of the yeast mannose-6-phosphate receptor homology (MRH) domain-containing protein YOS9 is, together with SEL1L, a cofactor of the HRD1 ubiquitin ligase complex, responsible for delivering misfolded glycoproteins like BRI1–5 or BRI1–9 to the degradation machinery in the ER membrane [7–10]. To investigate if OS9 and SEL1L are also involved in the disposal of SUBEX-C57Y, we ectopically expressed SUBEX-C57Y-GFP in *A. thaliana* wild-type and *os9-1* and *sel1l* knockout mutants. In a cycloheximide time-course experiment, we assessed the stability of stably-expressed SUBEX-C57Y over 18 h. In wild-type, SUBEX-C57Y was rapidly degraded and almost undetectable after 1 h (Figure 5A). In accordance with data from the transient-expression approach, SUBEX-C57Y degradation could be blocked by kifunensine (Figure 3B). By contrast, stabilization of SUBEX-C57Y was observed during the chase in *os9-1* plants even without kifunensine (Figure 5A).

We also checked the possible involvement of the proteasome in SUBEX-C57Y disposal by treating *A. thaliana* wild-type expressing SUBEX-C57Y with MG132. As observed for *N. benthamiana*, treatment with the proteasome inhibitor did not result in considerable stabilization of SUBEX-C57Y (Figure 5B). Similar to *os9-1*, SUBEX-C57Y degradation was also blocked in *sel1l* mutants and no further increase in protein accumulation was detected in the presence of kifunensine (Figure 5C).

We recently reported that deficiency of both class I α-mannosidases MNS4 and MNS5 inhibits degradation of the ERAD substrate BRI1–5 [11]. To check if mannose trimming by MNS4/MNS5 is required for SUBEX-C57Y degradation, we expressed SUBEX-C57Y in the *mns4 mns5* double knockout mutant. Consistent with the data for BRI1–5, SUBEX-C57Y protein levels were stabilized when compared with expression in wild-type plants and kifunensine had no additional stabilizing effect on SUBEX-C57Y protein levels in *mns4 mns5* plants (Figure 5C).

To examine whether the stabilizing effect of kifunensine on SUBEX-C57Y in wild-type *A. thaliana* depends indeed on the presence of its *N*-glycans, we compared SUBEX-C57Y with the non-glycosylated variant NQ123. As expected, the kifunensine-dependent accumulation of SUBEX-C57Y NQ123 was completely abolished (Figure 5D). Collectively, these data show that OS9, SEL1L and MNS4/MNS5 play an important role in SUBEX-C57Y degradation, which is mediated by one of the SUBEX-C57Y *N*-glycans.

### SUBEX-C57Y interacts with components of the plant ERAD machinery

**Figure 6:**
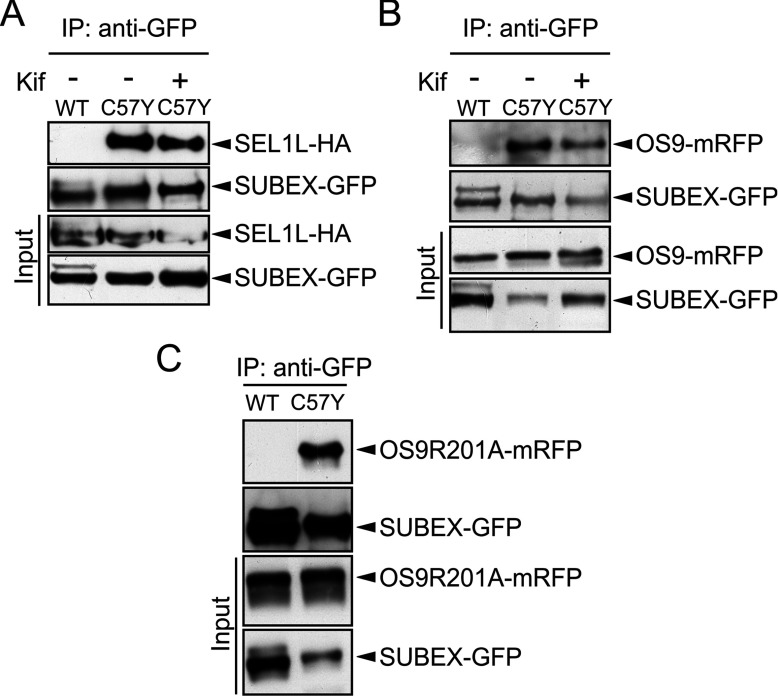
SEL1L and OS9 interact with SUBEX-C57Y independent of glycan trimming or a functional OS9 MRH domain (**A**) SUBEX-GFP and SUBEX-C57Y-GFP were transiently co-expressed with SEL1L-HA in *N. benthamiana*, in the presence or absence of 20 μM kifunensine (kif). (**B**) and (**C**) OS9-mRFP and OS9R201A-mRFP were co-expressed with SUBEX-GFP (WT) and SUBEX-C57Y-GFP (C57Y). SUBEX-GFP (WT) and SUBEX-C57Y-GFP (C57Y) were purified using GFP trap beads and co-purified proteins were analysed by immunoblotting with anti-HA or anti-mRFP antibodies. IP denotes the co-immunoprecipitated fraction.

Our results suggest that *N*-glycans from SUBEX-C57Y are trimmed by α-mannosidases and recognized by distinct proteins of the plant glycan-dependent ERAD complex. To investigate whether SUBEX-C57Y interacts with the HRD1 complex, members OS9 and SEL1L, we transiently co-expressed SUBEX-GFP and SUBEX-C57Y-GFP together with OS9-mRFP and SEL1L-HA in *N. benthamiana*, purified the GFP-tagged proteins by affinity chromatography and analysed the co-purification of OS9-mRFP and SEL1-HA by immunoblotting. In contrast with SUBEX-GFP, considerable co-purification of SUBEX-C57Y-GFP with SEL1L-HA (Figure 6A) or OS9-mRFP (Figure 6B) was observed. Treatment with kifunensine did neither abrogate the binding between SEL1L-HA and SUBEX-C57Y-GFP nor between OS9-mRFP and SUBEX-C57Y-GFP. We also tested the binding of SUBEX-C57Y to OS9R201A, a variant with a substitution in a highly conserved residue in the OS9 MRH domain [10]. SUBEX-C57Y could also be co-purified with OS9R201A (Figure 6C), indicating that SUBEX-C57Y interacts with OS9/SEL1L also in a glycan-independent manner.

### SUBEX-C57Y carries the terminal α1,6-mannose degradation signal

**Figure 7:**
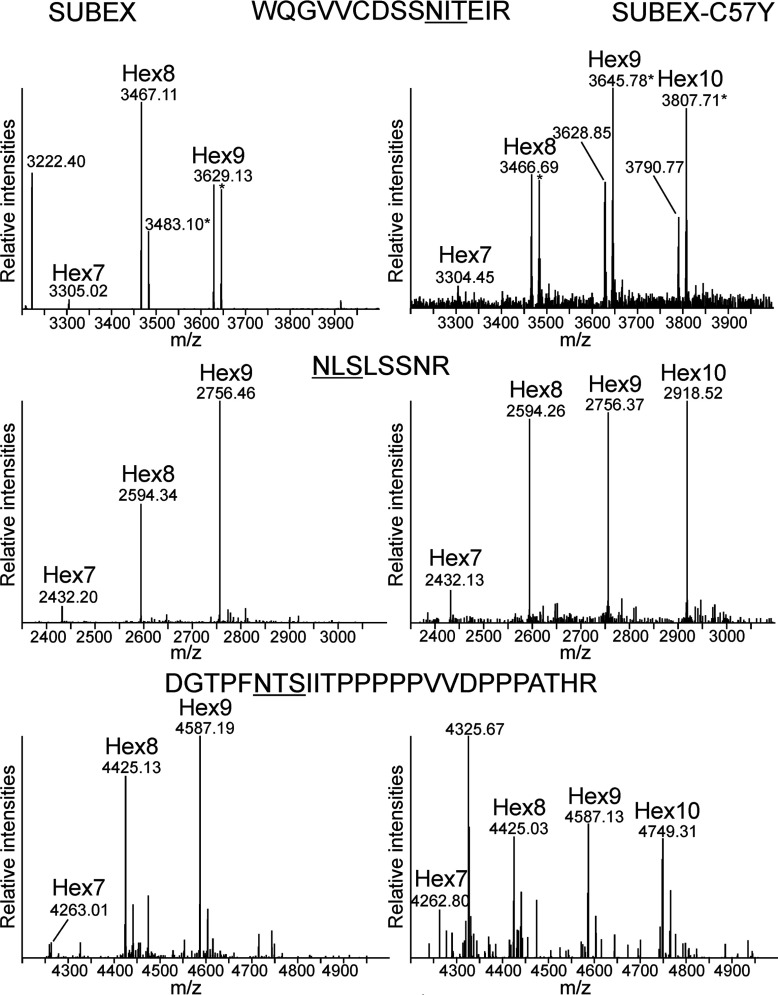
SUBEX and SUBEX-C57Y differ in their *N*-glycan structures SUBEX-GFP and SUBEX-C57Y-GFP were transiently expressed in *N. benthamiana*, purified, trypsin digested and the three glycopeptides analysed by LC-ESI-MS. The major peaks with masses corresponding to individual *N*-glycan structures are labelled. Hex7: Hex_7_HexNAc_2_; Hex8: Hex_8_HexNAc_2_; Hex9: Hex_9_HexNAc_2_; Hex10: Hex_10_HexNAc_2_. Ammonia adducts are indicated with an asterisk. The amino acid sequences of the peptides are indicated and the *N*-glycosylation sites are underlined.

In yeast and mammalian cells, an exposed α1,6-mannose residue present on *N*-glycans of ERAD substrates serves as a signal for degradation and is recognized by YOS9 or its human orthologues OS-9/XTP3-B [14,34,35]. In *A. thaliana*, MNS4 and MNS5 very likely generate this *N*-glycan signal on misfolded glycoproteins that is subsequently recognized by OS9 [9–11]. To identify the glycan-degradation signal on SUBEX-C57Y, we compared the *N*-glycan structures from SUBEX with SUBEX-C57Y. To this end, we expressed GFP-tagged versions of both proteins in *N. benthamiana* and subjected the purified proteins to LC-ESI-MS analysis. All three glycopeptides derived from SUBEX harboured very similar *N*-glycan structures. For each glycopeptide, the two major peaks correspond to peptides with Hex_9_HexNAc_2_ and Hex_8_HexNAc_2_ oligosaccharides, which are very likely derived from Man_9_GlcNAc_2_ and Man_8_GlcNAc_2_ *N*-glycans (Figure 7). On the other hand, SUBEX-C57Y contained considerable amounts of an additional peak corresponding to Hex_10_HexNAc_2_.

**Figure 8:**
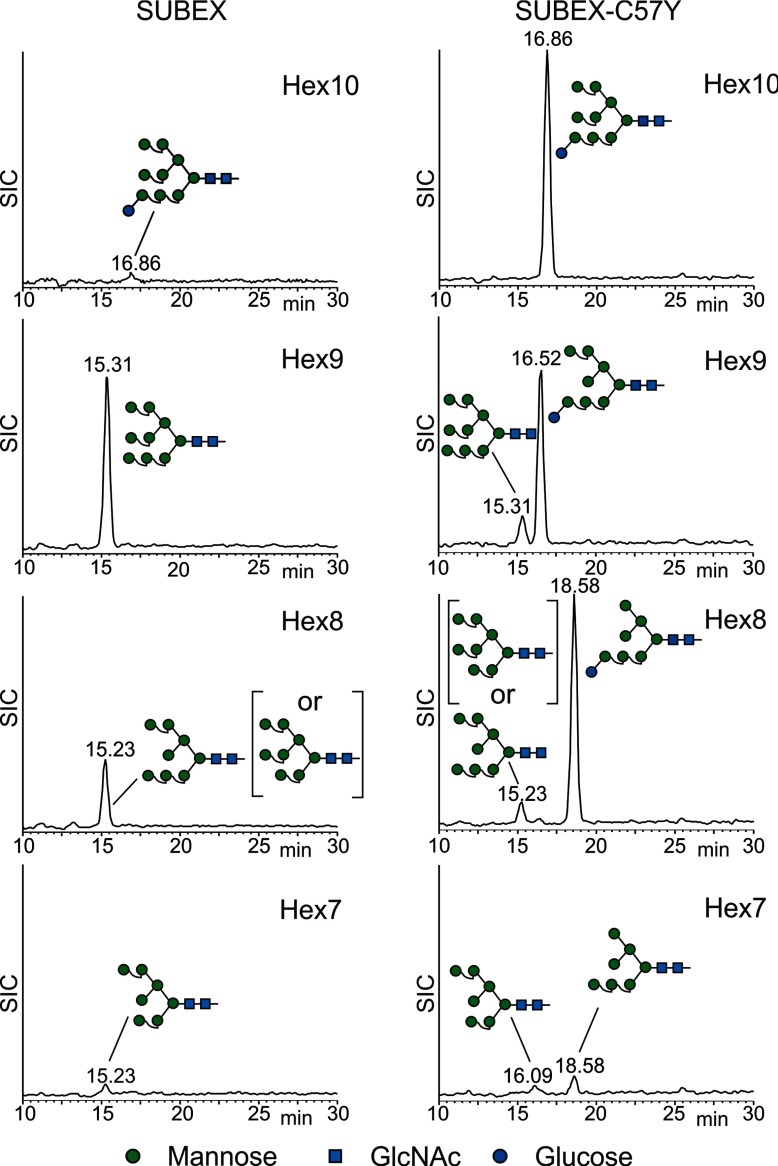
SUBEX-C57Y carries increased amounts of *N*-glycans with a free α1,6-linked mannose residue at the C-branch SUBEX-GFP and SUBEX-C57Y-GFP were transiently expressed in *N. benthamiana*, purified and the *N*-glycans were liberated and analysed by PGC-LC-ESI MS. Selected ion chromatograms (SICs) are shown. The assignment of structural isomers was based on reference glycans.

To further investigate the structures of the observed *N*-glycans, we performed isomeric analysis of released oligosaccharides by PGC-LC-ESI-MS [29]. By comparison of elution positions from standards, the indicated Hex_9_HexNAc_2_, Hex_8_HexNAc_2_ and Hex_7_HexNAc_2_ SUBEX peaks were confirmed as Man_9_GlcNAc_2_, Man_8_GlcNAc_2_ and Man_7_GlcNAc_2_ respectively and the Hex_10_HexNAc peak from SUBEX-C57Y was identified as Glc_1_Man_9_GlcNAc_2_. In contrast with SUBEX, only a small portion of the Hex_9_HexNAc_2_ and Hex_8_HexNAc_2_ peaks from SUBEX-C57Y were identified as Man_9_GlcNAc_2_ and Man_8_GlcNAc_2_ *N*-glycans. Instead, predominantly monoglucosylated Glc_1_Man_8_GlcNAc_2_ and Glc_1_Man_7_GlcNAc_2_ structures were detected (Figure 8). Consistent with our hypothesis, the Glc_1_Man_7_GlcNAc_2_ *N*-glycan displayed the free terminal α1,6-linked mannose residue on the C-branch, which most likely represents the *N*-glycan signal for SUBEX-C57Y degradation in plants.

## DISCUSSION

Given the large number of proteins being synthesized in the ER, it is of pivotal importance to precisely control their folding and maturation and eventually deliver aberrant proteins for degradation by different ERAD pathways. A deeper understanding of *N*-glycan-dependent ERAD processes in plants has so far been limited by the lack of appropriate substrates that allow dissection of the recognition, partitioning and clearance steps. In the present study, we investigated the fate of the ER-retained glycoprotein SUBEX-C57Y, which is degraded in a glycan-dependent manner in plants and interacts with components of the HRD1–ubiquitin ligase complex. The presence of only three *N*-glycans makes SUBEX-C57Y a very suitable glycoprotein substrate to investigate the contribution of the *N*-glycan position and the oligosaccharide structures to ERAD.

The block of SUBEX-C57Y degradation by the specific class I α-mannosidase inhibitor kifunensine demonstrates that mannose trimming of *N*-glycans in the ER plays a crucial role in the disposal of misfolded glycoproteins. The primary targets of ERAD inhibition by kifunensine in plants are the recently discovered ER α-mannosidases MNS4 and MNS5 that accelerate the generation of the free α1,6-linked mannose on the C-branch of *N*-glycans. This *N*-glycan acts very likely also as a degradation signal in plants [11,36] and, consistent with the proposed function of MNS4/MNS5, SUBEX-C57Y is stabilized in *mns4 mns5* knockout plants.

Interestingly, the proteasome inhibitors did not alter the SUBEX-C57Y protein levels, suggesting that the misfolded protein is disposed of via an alternative non-proteasome-dependent route. Whereas most yeast and mammalian ERAD substrates analysed so far are degraded via the cytosolic ubiquitin–proteasome system, several misfolded proteins appear also to be cleared in a proteasome-independent manner involving, for example, autophagy and delivery to the vacuole [37]. BRI1–9 degradation in *A. thaliana* was proteasome-dependent, but BRI1–5 disposal could not be blocked in the same way by MG132 treatment [18,19]. Two distinct degradation pathways were also observed for ricin A and B chains when expressed in tobacco protoplasts [38]. In line with findings for SUBEX-C57Y, these data highlight that different ERAD substrates are also cleared by distinct routes in plants. However, the specific intrinsic features that select protein substrates for distinct pathways, as well as the disposal route for SUBEX-C57Y, needs to be further investigated in the future.

In contrast with the well-known yeast ERAD substrate CPY* [32,33], none of the three N-glycans on SUBEX-C57Y denotes a context-specific degradation determinant. Independent of its position, the presence of a single *N*-glycan is sufficient to render SUBEX-C57Y a glycan-dependent ERAD substrate. Our data indicate a direct involvement of OS9 and SEL1L in the degradation of the misfolded SUBEX-C57Y protein. Protein–protein interaction between SUBEX-C57Y and OS9/SEL1L persisted in the presence of kifunensine or when the OS9R201A variant, which has a substitution of a conserved amino acid involved in mannose binding, was co-expressed. For *A. thaliana*, OS9 and its yeast and mammalian orthologues, binding to either non-glycosylated or glycosylated ERAD substrates with untrimmed oligomannosidic *N*-glycans has been shown in previous studies [10,39–41]. Similarly, OS9 may recognize a bipartite signal on misfolded proteins, consisting of the free α1,6-mannose on the C-branch of the *N*-glycan and an exposed non-native polypeptide region. Alternatively, since OS9 and SEL1L form a complex in plants and SEL1L orthologues can bind to misfolded proteins in a glycan-independent manner [10,40,42,43], OS9 and SEL1L might together decode the bipartite signal and associate with SUBEX-C57Y. In addition, other ER-resident proteins like binding protein (BiP) or PDIs that form a multi-protein complex with the ERAD factors in other organisms might play a role in recognition of the non-native protein determinant [44]. The cysteine at position 57 of SUBEX is thought to form an intramolecular disulfide-bond with Cys_66_, which presumably is important for folding or stabilization of the capping domain [22]. Consequently, the putative free thiol caused by the C57Y mutation could recruit PDI or another oxidoreductase, leading to changes in processing of *N*-glycans on misfolded proteins like SUBEX-C57Y. For example, yeast PDI1 forms a complex with the yeast MNS4/MNS5 homologue HTM1 that plays a role as folding sensor and directs the mannose trimming activity of HTM1 towards misfolded ERAD substrates [45]. A similar mechanism might be functional in plants, but a role of PDI family members or related proteins like ERdj5 [46] in ERAD of misfolded plant glycoproteins remains to be shown.

Isomeric analysis of *N*-glycans from SUBEX-C57Y confirmed the presence of increased levels of oligosaccharides with the exposed free α1,6-linked mannose residue on the C-branch. Importantly, SUBEX, which is also retained in the ER, does not display *N*-glycans carrying terminal α1,6-linked mannose residues. Consequently, our findings highlight that misfolding and generation of the *N*-glycan signal are interconnected. In contrast with SUBEX, where the dominant *N*-glycan structure was Man_9_GlcNAc_2_, considerable amounts of *N*-glycans on SUBEX-C57Y carried an additional single glucose residue on their A-branch. The present finding is not entirely surprising as the re-addition of a terminal glucose by UDP–glucose:glycoprotein glucosyltransferase and retention in the CNX/CRT-cycle is a well-known mechanism to keep un- or partially-folded proteins in a folding-competent state [47]. The presence of monoglucosylated *N*-glycans with terminal α1,6-mannose residues (Glc_1_Man_7_GlcNAc_2_) indicates that the folding and degradation pathways work tightly together. We purified SUBEX-C57Y from wild-type plants where the glycan-dependent ERAD pathway is active. As a consequence, the protein carrying Glc_1_Man_7_GlcNAc_2_ may represent the fraction that is still bound by CNX/CRT. The release from this ERQC cycle by glucosidase II catalysed deglucosylation could result in immediate clearance and, therefore, almost undetectable levels of SUBEX-C57Y with only Man_7_GlcNAc_2_ *N*-glycans. Alternatively, demannosylation on the B- and C-branches could reduce the reactivity of glucosidase II leading to higher amounts of monoglucosylated *N*-glycans on the misfolded protein that is subjected to degradation [48]. The distinct mannose removal from *N*-glycans can also provide a mechanism to prolong the interaction of incompletely folded monoglucosylated proteins with CNX/CRT and consequently to an increased chance of proper folding [49,50]. However, after several rounds of futile folding attempts, the protein has to be released and directed to ERAD. In mammals, the MNS4/MNS5 homologue, EDEM1, plays an important role in the termination of unproductive folding cycles [51]. In plants, further studies are required to unambiguously define the role of MNS4/MNS5 and the mannose trimming reactions in termination of folding cycles and delivery to ERAD. Together, our data show that SUBEX-C57Y is a novel ERAD substrate well-suited for defining the mechanism of glycan-dependent degradation in plants.

## References

[1] Ellgaard L., Helenius A. (2003). Quality control in the endoplasmic reticulum. Nat. Rev. Mol. Cell Biol..

[2] Hegde R. S., Ploegh H. L. (2010). Quality and quantity control at the endoplasmic reticulum. Curr. Opin. Cell Biol..

[3] Smith M. H., Ploegh H. L., Weissman J. S. (2011). Road to ruin: targeting proteins for degradation in the endoplasmic reticulum. Science.

[4] Howell S. H. (2013). Endoplasmic reticulum stress responses in plants. Annu. Rev. Plant Biol..

[5] Tintor N., Saijo Y. (2014). ER-mediated control for abundance, quality, and signaling of transmembrane immune receptors in plants. Front. Plant Sci..

[6] Verchot J. (2014). The ER quality control and ER associated degradation machineries are vital for viral pathogenesis. Front. Plant Sci..

[7] Liu L., Cui F., Li Q., Yin B., Zhang H., Lin B., Wu Y., Xia R., Tang S., Xie Q. (2011). The endoplasmic reticulum-associated degradation is necessary for plant salt tolerance. Cell Res..

[8] Su W., Liu Y., Xia Y., Hong Z., Li J. (2011). Conserved endoplasmic reticulum-associated degradation system to eliminate mutated receptor-like kinases in *Arabidopsis*. Proc. Natl. Acad. Sci. U.S.A..

[9] Su W., Liu Y., Xia Y., Hong Z., Li J. (2012). The *Arabidopsis* homolog of the mammalian OS-9 protein plays a key role in the endoplasmic reticulum-associated degradation of misfolded receptor-like kinases. Mol. Plant.

[10] Hüttner S., Veit C., Schoberer J., Grass J., Strasser R. (2012). Unraveling the function of *Arabidopsis thaliana* OS9 in the endoplasmic reticulum-associated degradation of glycoproteins. Plant Mol. Biol..

[11] Hüttner S., Veit C., Vavra U., Schoberer J., Liebminger E., Maresch D., Grass J., Altmann F., Mach L., Strasser R. (2014). *Arabidopsis* class I α-mannosidases MNS4 and MNS5 are involved in ER-associated degradation of misfolded glycoproteins. Plant Cell.

[12] Aebi M. (2013). N-linked protein glycosylation in the ER. Biochim. Biophys. Acta.

[13] Hebert D. N., Molinari M. (2012). Flagging and docking: dual roles for N-glycans in protein quality control and cellular proteostasis. Trends Biochem. Sci..

[14] Clerc S., Hirsch C., Oggier D., Deprez P., Jakob C., Sommer T., Aebi M. (2009). Htm1 protein generates the N-glycan signal for glycoprotein degradation in the endoplasmic reticulum. J. Cell Biol..

[15] Kanehara K., Xie W., Ng D. T. (2010). Modularity of the Hrd1 ERAD complex underlies its diverse client range. J. Cell Biol..

[16] Di Cola A., Frigerio L., Lord J., Roberts L., Ceriotti A. (2005). Endoplasmic reticulum-associated degradation of ricin A chain has unique and plant-specific features. Plant Physiol..

[17] Marshall R. S., Jolliffe N. A., Ceriotti A., Snowden C. J., Lord J. M., Frigerio L., Roberts L. M. (2008). The role of CDC48 in the retro-translocation of non-ubiquitinated toxin substrates in plant cells. J. Biol. Chem..

[18] Hong Z., Jin H., Tzfira T., Li J. (2008). Multiple mechanism-mediated retention of a defective brassinosteroid receptor in the endoplasmic reticulum of *Arabidopsis*. Plant Cell.

[19] Hong Z., Jin H., Fitchette A., Xia Y., Monk A., Faye L., Li J. (2009). Mutations of an alpha1,6 mannosyltransferase inhibit endoplasmic reticulum-associated degradation of defective brassinosteroid receptors in *Arabidopsis*. Plant Cell.

[20] Li J., Chory J. (1997). A putative leucine-rich repeat receptor kinase involved in brassinosteroid signal transduction. Cell.

[21] Chevalier D., Batoux M., Fulton L., Pfister K., Yadav R. K., Schellenberg M., Schneitz K. (2005). STRUBBELIG defines a receptor kinase-mediated signaling pathway regulating organ development in *Arabidopsis*. Proc. Natl. Acad. Sci. U.S.A..

[22] Vaddepalli P., Fulton L., Batoux M., Yadav R. K., Schneitz K. (2011). Structure-function analysis of STRUBBELIG, an *Arabidopsis* atypical receptor-like kinase involved in tissue morphogenesis. PLoS One.

[23] Liebminger E., Hüttner S., Vavra U., Fischl R., Schoberer J., Grass J., Blaukopf C., Seifert G., Altmann F., Mach L., Strasser R. (2009). Class I alpha-mannosidases are required for *N*-glycan processing and root development in *Arabidopsis thaliana*. Plant Cell.

[24] Schoberer J., Vavra U., Stadlmann J., Hawes C., Mach L., Steinkellner H., Strasser R. (2009). Arginine/lysine residues in the cytoplasmic tail promote ER export of plant glycosylation enzymes. Traffic.

[25] Farid A., Malinovsky F. G., Veit C., Schoberer J., Zipfel C., Strasser R. (2013). Specialized roles of the conserved subunit OST3/6 of the oligosaccharyltransferase complex in innate immunity and tolerance to abiotic stresses. Plant Physiol..

[26] Schoberer J., Liebminger E., Botchway S. W., Strasser R., Hawes C. (2013). Time-resolved fluorescence imaging reveals differential interactions of *N*-glycan processing enzymes across the Golgi stack in planta. Plant Physiol..

[27] Farid A., Pabst M., Schoberer J., Altmann F., Glössl J., Strasser R. (2011). *Arabidopsis thaliana* alpha1,2-glucosyltransferase (ALG10) is required for efficient *N*-glycosylation and leaf growth. Plant J..

[28] Stadlmann J., Pabst M., Kolarich D., Kunert R., Altmann F. (2008). Analysis of immunoglobulin glycosylation by LC-ESI-MS of glycopeptides and oligosaccharides. Proteomics.

[29] Pabst M., Grass J., Toegel S., Liebminger E., Strasser R., Altmann F. (2012). Isomeric analysis of oligomannosidic *N*-glycans and their dolichol-linked precursors. Glycobiology.

[30] Tretter V., Altmann F., März L. (1991). Peptide-N4-(*N*-acetyl-beta-glucosaminyl)asparagine amidase F cannot release glycans with fucose attached alpha 1-3 to the asparagine-linked *N*-acetylglucosamine residue. Eur. J. Biochem..

[31] Strasser R., Stadlmann J., Schähs M., Stiegler G., Quendler H., Mach L., Glössl J., Weterings K., Pabst M., Steinkellner H. (2008). Generation of glyco-engineered *Nicotiana benthamiana* for the production of monoclonal antibodies with a homogeneous human-like *N*-glycan structure. Plant Biotechnol. J..

[32] Kostova Z., Wolf D. H. (2005). Importance of carbohydrate positioning in the recognition of mutated CPY for ER-associated degradation. J. Cell Sci..

[33] Spear E. D., Ng D. T. (2005). Single, context-specific glycans can target misfolded glycoproteins for ER-associated degradation. J. Cell Biol..

[34] Quan E., Kamiya Y., Kamiya D., Denic V., Weibezahn J., Kato K., Weissman J. (2008). Defining the glycan destruction signal for endoplasmic reticulum-associated degradation. Mol. Cell.

[35] Hosokawa N., Kamiya Y., Kamiya D., Kato K., Nagata K. (2009). Human OS-9, a lectin required for glycoprotein endoplasmic reticulum-associated degradation, recognizes mannose-trimmed *N*-glycans. J. Biol. Chem..

[36] Hong Z., Kajiura H., Su W., Jin H., Kimura A., Fujiyama K., Li J. (2012). Evolutionarily conserved glycan signal to degrade aberrant brassinosteroid receptors in *Arabidopsis*. Proc. Natl. Acad. Sci. U.S.A..

[37] Schmitz A., Herzog V. (2004). Endoplasmic reticulum-associated degradation: exceptions to the rule. Eur. J. Cell Biol..

[38] Chamberlain K. L., Marshall R. S., Jolliffe N. A., Frigerio L., Ceriotti A., Lord J. M., Roberts L. M. (2008). Ricin B chain targeted to the endoplasmic reticulum of tobacco protoplasts is degraded by a CDC48- and vacuole-independent mechanism. J. Biol. Chem..

[39] Bhamidipati A., Denic V., Quan E. M., Weissman J. S. (2005). Exploration of the topological requirements of ERAD identifies Yos9p as a lectin sensor of misfolded glycoproteins in the ER lumen. Mol. Cell.

[40] Bernasconi R., Pertel T., Luban J., Molinari M. (2008). A dual task for the Xbp1-responsive OS-9 variants in the mammalian endoplasmic reticulum: inhibiting secretion of misfolded protein conformers and enhancing their disposal. J. Biol. Chem..

[41] Jaenicke L. A., Brendebach H., Selbach M., Hirsch C. (2011). Yos9p assists in the degradation of certain nonglycosylated proteins from the endoplasmic reticulum. Mol. Biol. Cell.

[42] Denic V., Quan E. M., Weissman J. S. (2006). A luminal surveillance complex that selects misfolded glycoproteins for ER-associated degradation. Cell.

[43] Christianson J. C., Shaler T. A., Tyler R. E., Kopito R. R. (2008). OS-9 and GRP94 deliver mutant alpha1-antitrypsin to the Hrd1-SEL1L ubiquitin ligase complex for ERAD. Nat. Cell Biol..

[44] Ushioda R., Hoseki J., Nagata K. (2013). Glycosylation-independent ERAD pathway serves as a backup system under ER stress. Mol. Biol. Cell.

[45] Gauss R., Kanehara K., Carvalho P., Ng D. T., Aebi M. (2011). A complex of Pdi1p and the mannosidase Htm1p initiates clearance of unfolded glycoproteins from the endoplasmic reticulum. Mol. Cell.

[46] Ushioda R., Hoseki J., Araki K., Jansen G., Thomas D. Y., Nagata K. (2008). ERdj5 is required as a disulfide reductase for degradation of misfolded proteins in the ER. Science.

[47] D’Alessio C., Caramelo J. J., Parodi A. J. (2010). UDP-Glc:glycoprotein glucosyltransferase-glucosidase II, the ying-yang of the ER quality control. Semin. Cell Dev. Biol..

[48] Ito Y., Takeda Y. (2013). Deciphering the roles of glycan processing in glycoprotein quality control through organic synthesis. Biosci. Biotechnol. Biochem..

[49] Cabral C., Choudhury P., Liu Y., Sifers R. (2000). Processing by endoplasmic reticulum mannosidases partitions a secretion-impaired glycoprotein into distinct disposal pathways. J. Biol. Chem..

[50] Stigliano I. D., Alculumbre S. G., Labriola C. A., Parodi A. J., D’Alessio C. (2011). Glucosidase II and N-glycan mannose content regulate the half-lives of monoglucosylated species *in vivo*. Mol. Biol. Cell.

[51] Molinari M., Calanca V., Galli C., Lucca P., Paganetti P. (2003). Role of EDEM in the release of misfolded glycoproteins from the calnexin cycle. Science.

